# The Role of Conspiracy Theories, Perceived Risk, and Trust in Science on COVID-19 Vaccination Decisiveness: Evidence from Cyprus

**DOI:** 10.3390/ijerph20042898

**Published:** 2023-02-07

**Authors:** Marilena Mousoulidou, Andri Christodoulou, Michailina Siakalli, Marios Argyrides

**Affiliations:** Department of Psychology, Neapolis University Pafos, Paphos 8042, Cyprus

**Keywords:** COVID-19, pandemic, vaccination, conspiracy theories, trust in science, perceived risk, social media

## Abstract

COVID-19 reminded us of the importance of vaccinating for successfully overcoming health-related crises. Yet, vaccine hesitancy is still present. This study examined the impacts of conspiracy theories, perceived risk, and trust in science on COVID-19 vaccination decisiveness. The study was conducted at the end of the third wave of the pandemic, in July 2021, in Cyprus. Data were collected via an online self-administered anonymous survey using convenience and snowball sampling methods. Participants were 363 adults who completed a set of questionnaires that examined their believability in ten vaccine-related conspiracy theories, their perceived dangerousness of COVID-19, and their level of trust in science and scientists. The results suggest that (a) participants with a high conspiracy theory belief are less likely to be vaccinated, (b) participants who perceive COVID-19 as a dangerous disease are more likely to be vaccinated, and (c) participants with high trust in science are more likely to be vaccinated. The implications of the findings are discussed and can be used by public health officials in their campaigns.

## 1. Introduction

More than two and a half years have passed since the time the World Health Organization (WHO) declared COVID-19 as a pandemic in March 2020 [1]. Globally, as of 18 September 2022, more than 609 million confirmed cases and over 6.5 million related confirmed deaths have been reported [2]. The pandemic seriously affected the healthcare systems worldwide and especially healthcare professionals from countries that were heavily hit by the pandemic, who experienced longer working hours, higher workloads, and frequent on-call shifts, among others [3,4,5]. Unavoidably, this impacted the well-being of this population, with research suggesting that they experienced emotional exhaustion and a higher risk for burnout [3,4]. The impact of the pandemic on the mental health of the general population is also a well-documented finding [6,7,8,9,10,11,12,13,14]. Vaccination is vital for successfully alleviating the problems associated with the spread of the virus and combating the pandemic [15]. As of 11 December 2020, the U.S. Food and Drug Administration (FDA) issued an emergency use authorization for the first COVID-19 vaccine for individuals older than 16 years of age [16], followed by a second vaccine on December 18 [17], and a third on 27 February 2021 [18]. Additional COVID-19 vaccines, including viral vaccines, were introduced in 2021 [19], and as of December 2022, 11 COVID-19 vaccines were granted Emergency Use Listing (EUL) by the WHO [20]. Nevertheless, even though vaccination against COVID-19 is available, a widespread hesitancy from the population toward receiving it has been well-documented in the literature [21,22,23,24,25]. This has been a particular challenge for health authorities in their attempt to find ways to increase the populations’ vaccination uptakes.

The problem with vaccination hesitancy is not new. A general refusal or delay in the acceptance of vaccines despite their availability, such as vaccines for childhood illnesses or other diseases (e.g., human papillomavirus and influenza), has been documented long before the COVID-19 pandemic [26,27,28]. This “forced” the WHO to declare vaccination hesitancy as one of the ten major global health threats for 2019 [29]. The reasons behind vaccination decisiveness have been extensively studied in the literature, and many reasons for individuals’ refusal to vaccinate have been identified. More specifically, vaccine hesitancy is defined as a behavior influenced by issues of confidence, complacency, and convenience whereby individuals make vaccine-specific decisions based on political, religious/personal, and scientific determinants (see [27] for more details).

A significant contributor to vaccine hesitancy is the information accessible to the public. The COVID-19 pandemic was a sudden major crisis, and with the uncertainty surrounding the transmissibility and the progression of the virus, people urgently searched for health information to protect themselves and their families and friends. Much of the information garnered came from online sources and social platforms, characterizing the accessibility and efficiency provided by technological advancements. This, however, poses the challenge of discerning between credible and unsubstantiated sources. During periods of uncertainty and fear, misinformation exacerbates [30,31,32]. The COVID-19 pandemic was not an exception to this. With the help of social media, from early on, misinformation and conspiracy theories regarding the pandemic started to circulate [30,33]. Some of this misinformation focused on the vaccine, with a popular conspiracy theory claiming that the mRNA technology used in the vaccine could alter people’s DNA [34,35]. Research has shown that conspiracy theories influence vaccination decisiveness leading to a lower acceptance of the vaccination [36,37,38]. Therefore, (mis)information has been related to vaccine hesitancy and the perceived risk of the virus [39,40]. How or where one accesses information can impact whether one perceives the virus as dangerous and how willing one is to get vaccinated [41].

Perceived risk of the pandemic could also influence individuals’ intentions to get vaccinated. People who perceive high levels of risk from COVID-19 are more likely to get vaccinated against the virus than those who perceive a low level of risk [42,43,44]. For instance, one study has shown that the perceived severity of the virus was one of the significant predictors of vaccination intent [45]. In fact, several studies linked risk perception with hesitancy or willingness to get vaccinated against the SARS-CoV-2 virus [40,46,47,48,49].

Trust in science has also been identified as a factor influencing people’s decisions, such as vaccination decisiveness, during major health crises and periods of uncertainty. This is not a surprise considering that scientists are the ones who recommend how to protect oneself from the virus (e.g., recommended public health measures, vaccination, etc.). The literature that examined the relationship between trust in science and vaccination hesitancy in the COVID-19 era has shown that individuals who have high trust in science are more likely to accept a COVID-19 vaccine [42,50,51,52], and those who have lower trust in science are more likely to refuse to get vaccinated [53,54]. Importantly, research has also shown that trust in science is negatively related to misinformation and conspiracy theories. People who show a lower level of trust in science and trust in the government are more likely to believe misinformation and conspiracy theories that present vaccines as dangerous or unnecessary [54,55,56]. This also appears to be related to social media. People who spend more time on social media can more easily find vaccine misinformation and conspiracy theories that might influence their willingness to vaccinate [54,57,58].

The present study took place in Cyprus. In Cyprus, the first COVID-19-positive cases were reported on the 9th of March 2020 [59], and by 12 March 2020, the government issued a formal announcement regarding the number of confirmed cases [60]. The first COVID-19-related death was reported on 21 March 2020 [59]. A few days after the first COVID-19 vaccine was approved by FDA, on 15 December 2020, the government of Cyprus issued and presented its vaccination plan [61]. This included the development of 38 vaccination centers, mobile units that would vaccinate priority groups on the spot, and the possibility of up to 100 different units operating simultaneously [61]. The vaccine was available to the population incrementally. Priority was first given to staff and residents of facilities for the elderly and chronically ill, followed by healthcare professionals within hospital settings, then individuals belonging to vulnerable groups, followed by healthcare professionals, personnel, and residents of closed structures (e.g., prisons). Lastly, the vaccine was available to the rest of the population according to age [62]. To date, no vaccine mandates have been applied.

To our knowledge, there are a few studies that examined vaccine hesitancy in the population of Cyprus. Kyprianidou et al.’s [63] cross-sectional research was conducted a few months before the current study (December 2020 and January 2021) and examined the profiling of COVID-19 vaccination hesitancy in individuals from six European countries. Results from 832 participants showed that hesitancy toward COVID-19 vaccination is a complex phenomenon with sociodemographic (age, place of residency, having under-aged children) and behavioral/attitudinal factors (compliance with protective measures, institutional trust, tiredness toward COVID-19) influencing vaccine acceptance. Cyprus, which is of great importance for the current study, had the highest rates of COVID-19 vaccination hesitancy compared to Poland, Italy, and Spain. The authors suggested that this could be related to the higher pandemic fatigue (tiredness toward COVID-19) and lower institutional trust that was observed in Cyprus [63]. Another study that was conducted in Cyprus examined the relationship between conspiracy theories circulating in social media, trust in science, and compliance with protective measures [64]. This study examined Greek and Cypriot citizens and was conducted during the first wave of the pandemic (April 2020). Even though this research did not examine vaccination decisiveness, it is nevertheless important since it provided some insight into the characteristics of people who believe in conspiracy theories. Their results showed that believability in conspiracy theories was more strongly related to people who are less educated, are of younger age, have lower incomes, live in less densely populated areas, and have lower trust in science, which leads to less compliance with the protective measures. Additionally, feelings of sadness, helplessness, distress, and being on edge were also strong predictors of believability in conspiracy theories [64].

The period that the current study was conducted can be considered the end of the third wave of the pandemic; June and July 2021. This period is believed to be important for two reasons. First, the SARS-CoV-2 virus vaccines (Pfizer/BioNTech, AstraZeneca, Moderna, and Johnson & Johnson) were available nationally to people over the age of 18 [65]. Second, during that period, the government implemented the “SafePass” measure to encourage vaccination rates [66]. The “SafePass” denoted that the individual was vaccinated, recently recovered from COVID-19, or underwent a recent rapid antigen test or PCR [67]. This measure was mandatory for indoor and outdoor spaces where the risk of transmission was increased (e.g., clubs, bars, and workplaces) [67]. Up until 19 June 2021, 696,317 doses were administered, with 46.9% of the population receiving at least one dose of the vaccine [68], and by 1 July 2021, this percentage reached 65% [69].

The aim of the current study was to examine the relationship between vaccination decisiveness and the variables (a) conspiracy theories, (b) trust in science, and (c) perceived risk of the SARS-CoV-2 virus in the population of Cyprus. Based on this aim and the existing literature, the following hypotheses were formulated:

1. There will be a negative correlation between vaccination decisiveness and conspiracy theories. Based on previous literature, we expected that individuals with high believability in conspiracy theories would be less likely to be vaccinated.
2. There will be a positive association between vaccination decisiveness and the perceived risk of the COVID-19 pandemic. In line with existing research, we hypothesized that those considering COVID-19 a dangerous disease would be more likely to be vaccinated.
3. There will be a positive correlation between vaccination decisiveness and trust in science. Previous findings suggest that individuals who have high trust in science are more likely to follow the mandatory guidelines and, thus, more likely to be vaccinated.
4. There will be associations between social media use and vaccination decisiveness, conspiracy theories, and trust in science. In line with previous findings, we expected social media to influence the aforementioned variables.
5. There will be associations between the demographics of participants and vaccination decisiveness. The literature suggests that specific demographics, such as age, education, and sex, influence vaccination decisiveness [63,70,71,72]. We explored whether such differences exist in the present sample.

## 2. Materials and Methods

### 2.1. Procedure

Upon necessary ethical approval from the Cyprus National Bioethics Committee (ΕΕΒΚ ΕΠ 2021.01.150), the measures used in this study were combined into one questionnaire created via Google Forms. Data were collected via convenience and snowball sampling methods between 23 June 2021 and 7 July 2021. The questionnaire was distributed to the participants online using email, social chatting apps (e.g., WhatsApp, Viber, Messenger), and social media (e.g., Facebook). All participants were contacted online, informed about the purpose and duration of the study, and assured of their anonymity in participating. Their consent to participate in the research was received before completing the questionnaire. The completion of the questionnaire took approximately 15 min.

### 2.2. Sample

A total of 363 participants clicked the link and completed the survey. All responses were included in the analysis. Due to the settings of Google Forms, there were no missing data. Eligibility for participation in this study included residents of Cyprus who were 18 years and over. There were no exclusion criteria.

### 2.3. Measures

Demographic information: A demographic questionnaire was administered, which asked participants to provide their sex, age, education level, and place of residency.

Specific questions to COVID-19: Three questions were developed and administered to examine the characteristics of our sample regarding COVID-19. Question 1 asked participants to state the most commonly used source of information about COVID-19. Participants were given eight options with the ability to select multiple answers. The options were: (a) social media (Facebook, Instagram, Twitter, etc.), (b) newspapers, (c) TV programs, (d) news bulletins, (e) the official website of Cyprus’s Ministry of Health, (f) official website of the World Health Organization, (g) doctors and other healthcare professionals, and (h) scientific journals. The second question asked participants to report whether they perceived COVID-19 as a dangerous disease. The participants had to select from the options: (a) it is not dangerous, (b) it is moderately dangerous, and (c) it is very dangerous. Question 3 was a yes-or-no question that asked participants whether they had been vaccinated against COVID-19 with at least the first dose of the vaccine.

Conspiracy Theory Questionnaire (CTQ): To measure participants’ believability of vaccine-related conspiracy theories, we used the CTQ [73]. This is a 17-item questionnaire in the Greek language that examines participants’ general believability of conspiracy theory statements. The questionnaire was developed by the authors based on two criteria: (a) the conspiracy theory statements were popular in Cyprus at the time of the study, and (b) the conspiracy theory statements were circulating in social media during the time the current study was conducted. For the current study, out of the 17 original items included in CTQ, 10 items that reflected vaccine-related statements were used. Reliability analysis of these 10 items of the CTQ indicated very good internal consistency with a Cronbach’s alpha value of 0.87. The readability level of the questionnaire was basic secondary school reading level and was piloted on a small convenience sample (*n* = 10) of the expected age groups of the study (young adults, middle-aged adults, and older adults). Based on this piloting, minor changes were made to the syntax of the statements. Each of the final 10 conspiracy theory statements used in the current study had to be rated on a 5-point Likert-type scale ranging from 1 (extremely unbelievable) to 5 (extremely believable).

Trust in science and scientists inventory: To explore the participants’ levels of trust in science and scientists, the Trust in Science and Scientists Inventory [74] ([75] for the Greek version) was employed. The inventory includes 21 items that measure the level of trust in science and scientists (e.g., I trust that the work of scientists is to make life better for people), with a higher score indicating a higher trust level. Responses are rated on a 5-point Likert-type scale ranging from 1 (strongly disagree) to 5 (strongly agree). The coefficient alpha for the current sample was very high, α = 0.9.

### 2.4. Statistical Analyses

All data were entered into the Statistical Package for Social Sciences, Version 25.0 (SPSS 25, IBM Corporation, Armonk, NY, USA), the level of significance for the tests was set to 5%, and all the necessary analyses were conducted. Descriptive statistics were computed. Dividing our sample based on vaccination status (vaccinated or not), we created three percentile groupings to assess the belief in each of the ten conspiracy theory statements. Subsequently, we conducted point-biserial correlations, Pearson correlations, *t*-tests, and chi-square tests to assess the association between vaccination decisiveness and the variables of interest. In addition, we conducted a logistic regression to examine the impact of the variables of interest and the likelihood of vaccination.

## 3. Results

The demographic characteristics of the participants are presented in Table 1. The sample comprised 363 participants aged 18–74 years. Of these, 267 (73.6%) were females, and 96 (26.4%) were males. Most of the participants lived in an urban setting (293; 80.7%) and had a Greek–Cypriot nationality (322; 88.7%). Moreover, 10.74% had a high school diploma, 37.74% had a bachelor’s degree, and 51.52% had master’s or/and doctorate degrees. Of the total sample, 120 participants (33.1%) were non-vaccinated and 243 (66.9%) were vaccinated. The age of the sample ranged from 18 to 74, with 80 participants (22.04%) being young adults (18–30 years old), 240 participants (66.12%) early–middle-aged adults (31–50 years old), and 43 participants (11.84%) late–middle-aged/older adults (51+ years old).

To explore the believability of our 363 participants to the 10 conspiracy theories, we divided our sample into 2 groups based on whether they were vaccinated or not. For each group, we then created 3 percentile groupings to evaluate their beliefs in each of the 10 conspiracy theories: (1) no-to-weak belief (1st to 25th percentile), (2) neutral belief (26th to 75th percentile), and (3) moderate-to-strong belief (76th to 100th percentile). As it is clear from Table 2, the two groups rated the ten conspiracy theories differently.

To test Hypothesis 1, a point-biserial correlation was used to determine the relationship between vaccination decisiveness and conspiracy theories. There was a statistically significant negative correlation between the two variables of interest (r = −0.470, *p* < 0.001), which supported Hypothesis 1. This implies that the higher the believability in conspiracy theories, the lower the possibility of being vaccinated. In addition, an independent samples *t*-test was conducted to explore possible statistically significant differences between vaccination decisiveness and conspiracy theories. Statistically significant differences were revealed for the variable conspiracy theories (t(361) = 10.12, *p* < 0.001), where non-vaccinated individuals had higher scores for the variable of interest (M = 27.49, SD = 7.52) than vaccinated individuals (M = 19.99, SD = 6.16).

A chi-square test for independence indicated a significant association between vaccination decisiveness and the perceived risk of the COVID-19 pandemic $\chi^{2}\left(2,\;363\right)=31.90,\;p<0.001,\;phi=0.3$. Vaccinated individuals were more likely to consider COVID-19 a dangerous disease than non-vaccinated individuals, supporting Hypothesis 2.

A point-biserial correlation indicated a significant positive association between vaccination decisiveness and trust in science (r = 0.36, *p* < 0.001). This implies that vaccinated individuals tend to have higher levels of trust in science than non-vaccinated individuals, which supports Hypothesis 3. Furthermore, a *t*-test was used to examine possible differences between the aforementioned variables of interest. The results revealed statistically significant differences for the variable trust in science (t(361) = −7.34, *p* < 0.001) where vaccinated individuals had higher mean scores (M = 75.84, SD = 11.74) than non-vaccinated individuals (M = 66.60, SD = 11.45). We also explored the relationship between conspiracy theories, and trust in science using the Pearson correlation. The analysis revealed a statistically significant negative association between the variables of interest (r = −0.725, *p* < 0.001). A higher level of trust in science was associated with lower levels of believability in conspiracy theories. To examine Hypothesis 4, we assessed the sources that individuals use to gather information about the pandemic. We found that the highest preferred source was social media (*n* = 264), followed by news bulletins (*n* = 223) and the official website of Cyprus’s Ministry of Health (*n* = 108). Newspapers (*n* = 97), doctors and other healthcare professionals (*n* = 89), and TV programs (*n* = 85) were reported at a lower rate. The sources reported less frequently included the official website of the World Health Organization (*n* = 63) and scientific journals (*n* = 43). Chi-square tests were applied to explore possible associations between participants’ most preferred sources of information (social media) and vaccination decisiveness. Of the 264 participants who used social media to gather information about the pandemic, 164 (62.1%) were vaccinated, and 100 (37.9%) were non-vaccinated. The chi-square test revealed a statistically significant association between the use of social media and vaccination decisiveness $\chi^{2}\left(1,\;363\right)=9.383,\;p=0.002,\;phi=-0.167$. The proportion of vaccinated and non-vaccinated individuals that use social media as a source of information for the pandemic is statistically different. The proportion of vaccinated people using social media is higher than the proportion of non-vaccinated individuals. Since news bulletins were also one of the participants’ preferred sources of information, a chi-square test was conducted. The test showed that there was no significant relationship between the use of news bulletins and vaccination decisiveness $\chi^{2}\left(1,\;363\right)=2.738,\;p=0.09,\;phi=0.093$. Furthermore, there were statistically significant differences between users and non-users of social media regarding conspiracy theory beliefs (t(361) = −3.71, *p* < 0.01). The mean value in conspiracy theory beliefs for users of social media (M = 23.22, SD = 7.75) was higher than the mean for non-users (M = 20.45, SD = 6.43). The same applies to the variable trust in science (t(361) = 4.109, *p* < 0.01), where users of social media reported lower mean values in trust in science (M = 71.23, SD = 12.12) as compared to non-users (M = 76.94, SD = 10.91).

Social media use with respect to conspiracy theory belief was explored. By restricting the sample to individuals that use social media, results showed that vaccinated and non-vaccinated individuals have statistically significant differences in conspiracy theories believability (t(261) = 8.15, *p* < 0.001). Namely, for non-vaccinated participants, the mean score in conspiracy theory belief is higher than that of vaccinated participants, i.e., M = 27.69, SD = 7.86 and M = 20.51, SD = 6.31.

We further computed a logistic regression to ascertain the impact of four independent variables (trust in science, conspiracy theory, social media, and perceived risk) on the likelihood that individuals will be vaccinated. The model was statistically significant $(\chi^{2}\left(5,\;363\right)=95.19,\;p<0.001).$ The model explained between 23% (Cox and Snell R square) and 32% (Nagelkerke R-squared) of the variance in vaccination status and correctly classified 76.3% of cases. Conspiracy theory beliefs made a unique statistically significant contribution to the model. Increased conspiracy belief was associated with a reduction in the likelihood of being vaccinated by 0.877 times. The trust in science, social media, and perceived risk were not associated with vaccination decisiveness. The logistic regression is presented in Table 3.

To test Hypothesis 5 chi-square tests were used to explore the relationship between vaccination decisiveness and the demographics of gender, educational level, and age. There was no significant association between gender and vaccination decisiveness $\left(\chi^{2}\left(2,\;363\right)=0.037,\;p=0.847,\;phi=-0.017\right)$. Furthermore, no significant relationship between educational level and vaccination decisiveness was found $\left(\chi^{2}\left(1,\;363\right)=3.879,\;p=0.144,\;phi=0.103\right)$. A significant association between age (young adults, early–middle-aged adults, and late–middle-aged/older adults) and vaccination decisiveness was revealed ($\chi^{2}\left(2,\;363\right)=25.652,\;p<0.001,\;phi=0.266$). Within the young adult age group, the proportion of vaccinated and non-vaccinated individuals differs significantly. Specifically, the number of non-vaccinated individuals was greater than that of vaccinated individuals. Within the early–middle-aged adults group, there was a statistically significant difference in vaccination decisiveness with the proportion of vaccinated early middle-aged adults being higher than non-vaccinated ones. There was no statistically significant difference in the proportion of vaccinated and non-vaccinated in the late–middle-aged/older adults group.

## 4. Discussion

Despite their availability, a general refusal or delay in the acceptance of vaccines has been well-documented in history [27,29]. The COVID-19 pandemic reminded us why vaccination is important. As the pandemic is still ongoing, behaviors protecting against the virus are very important, considering the underlying interactions of COVID-19 with other disorders have not yet been fully understood by the scientific community. The current study aimed to investigate vaccine decisiveness by examining the role of conspiracy theories, trust in science, perceived risk of the SARS-CoV-2 virus, and social media. The data were obtained in Cyprus after the end of the third wave of the COVID-19 pandemic in July 2021. Overall, our results suggest that vaccination acceptance is influenced by conspiracy theories, trust in science, perceived risk, and social media, with conspiracy theories being the most critical factor. The current study’s findings add to the existing literature examining the possible factors influencing individuals’ vaccine decisiveness.

First, our results show a negative relationship between vaccination decisiveness and conspiracy theories. Individuals who believe in conspiracy theories are less likely to be vaccinated. This finding is in accordance with the existing literature that suggests that conspiracy theories are related to a lower acceptance of the vaccination [36,37,38]. In addition, by dividing our sample based on their vaccination status, we were able to observe a different trend between vaccinated and non-vaccinated individuals regarding conspiracy theories. When the two groups are compared (see Table 1), the believability scores of non-vaccinated individuals are different from vaccinated individuals both in terms of moderate-to-strong belief (non-vaccinated: percentages ranging from 10–51.7%; vaccinated: percentages ranging from 1.6–14.4%), and no-to-weak belief (non-vaccinated: percentages ranging from 18.3–68.3%; vaccinated: percentages ranging from 42.8–95.1%). Importantly, however, even vaccinated individuals still believed or held a neutral stance toward some of the conspiracy theories. For instance, 21.4% of vaccinated individuals moderately or strongly believed that researchers rushed to develop the vaccine and, therefore, its effectiveness and safety cannot be trusted. Moreover, the neutral responses of vaccinated individuals for some conspiracy theories were as high as those of non-vaccinated individuals (non-vaccinated: highest neutral response 39.2%; vaccinated: highest neutral response 42.8%). In fact, for the conspiracy theory claiming that the vaccine could affect women’s fertility, the responses of vaccinated individuals were split between no-to-weak believability and neutral belief (42.8 for both). Even though, as a whole, the percentages of vaccinated individuals that held a neutral stance were lower than those of non-vaccinated individuals (non-vaccinated: percentages ranging from 21.7–39.2%; vaccinated: percentages ranging from 3.3–42.8%), the fact that both vaccinated and non-vaccinated individuals believe or are neutral with respect to conspiracy theories is alarming. The seriousness of this finding can be seen from the logistic regression analysis, which showed that believability in conspiracy theories was the factor that predicted vaccination decisiveness.

Second, we found that the perceived risk of COVID-19 is different between vaccinated and non-vaccinated individuals. Supporting the existing literature, our results show that vaccinated individuals perceived the COVID-19 pandemic as more dangerous than non-vaccinated individuals [40,42,43,44,45,47,48,49]. Perceived dangerousness of the disease might increase the fear of contracting a dangerous disease, which in turn might influence vaccination intention [47]. The logistic regression results indicate that risk perception is marginally statistically significant, which shows the importance of this variable in predicting vaccination intention.

Third, we found that social media plays its own role in vaccination intention. First, our results show that social media was the most preferred source to gather information about the pandemic. Second, when we examined for differences between vaccinated and non-vaccinated individuals that use social media, we found that those who were non-vaccinated had higher conspiracy theory beliefs than those who were vaccinated. This supports the literature suggesting that social media “promote” conspiracy theories and, thus, influence individuals’ willingness to get vaccinated [54,57,58].

Moreover, our study underlines the role of trust in science in vaccination decisiveness. The current results show that vaccinated individuals have higher trust in science than non-vaccinated individuals, which supports previous research findings [42,50,51,52,53,54,64]. Additionally, we also found that high trust in science is associated with a lower level of conspiracy theory belief [54,55,56]. It is clear that trusting science and scientists is vital for successfully overcoming critical periods. Not only because recommendations on how to combat the pandemic usually come from scientists, but also because trust in science can act as a protective factor against conspiracy theories.

Lastly, this study shows that age influences vaccination decisiveness. We found that the number of non-vaccinated young adults was greater than that of vaccinated young adults. These results were reversed for early–middle-aged adults, whereby vaccinated individuals were higher in numbers than non-vaccinated. For the current participants, we did not find any significant differences in gender and educational levels. This does not support the literature that suggests that gender and education are important for vaccination intention [64,70,71,72].

The current study has several practical implications. First and foremost, it is clear from the current results that conspiracy theory beliefs predict vaccine decisiveness. This clearly supports the importance of finding ways to combat the increased misinformation/conspiracy theories available, especially during crises. From the early stages of the pandemic, the WHO established the Information Network for Epidemics (EPI-WIN) [76]. EPI-WIN aims to give everyone access to accurate, timely, and easy-to-understand updated information about the development of the pandemic. The website provides information that comes from “trusted” sources with the goal of debunking myths and misinformation about the pandemic [76]. Additionally, the WHO and the CDC strive to help the public distinguish between valid information and conspiracy theories regarding the vaccine (see for example, vaccine safety by the WHO [77], and myths and facts about COVID-19 vaccines by CDC [34]). Despite these efforts by health agencies, the challenge remains. More campaigns are needed that will make people aware of the positive impacts of vaccinations and, at the same time, tackle conspiracy theories. These campaigns can also be utilized to increase the public’s perceived risk of the pandemic. Our results suggest that raising awareness of the disease’s dangerousness might improve vaccination uptake. Finding ways to build or restore the population’s trust in science during health crises is equally important. Education that improves information literacy and knowledge of scientific facts versus conspiracy theories will help in improving trust in science and scientists. Lastly, since social media is a common way to gather information about the pandemic, interventions aimed specifically at people that use social media should be implemented. This can involve campaigns that increase people’s awareness of the misinformation and conspiracy theories available on social media and highlight the vaccine’s usefulness. The major social media platforms, such as Facebook, Twitter, and YouTube, updated their policies in an attempt to increase awareness and reduce the misinformation available on their platforms [78,79,80]. These policies are continuously updated to combat new misinformation that appears as the pandemic progresses. Whether this technique works is still open for discussion, as research findings suggest that misinformation is still readily available to the public [81,82,83,84]. Similar strategies, such as fake news regulations, were also employed by governments around the world [85]. It is imperative to continue these efforts.

Though the current study provides useful information, it is not without its limitations. The generalizability of the findings is impeded due to the small sample size and the convenience sampling method implemented. Similarly, most of the participants were highly educated (tertiary education) and female, possibly introducing an overrepresentation of this population in the sample and thus resulting in bias. In addition, there was a disproportionate number of vaccinated (66.9%) versus non-vaccinated (33.1%) participants who might have affected our findings. Furthermore, other factors that may contribute to vaccine decisiveness were not explored in the current study. For instance, a recent study explored the role of religiosity on COVID-19 vaccination decisiveness in 90 countries [86]. They examined four religions and non-believers and found that religiosity and vaccination decisiveness was only linked with Christianity, with Christians being less likely to get vaccinated. Cyprus is a highly religious country, with more than 70% of the population being Christians [87]; however, the impact of religiosity on vaccine decisiveness was not explored in the current study. Future research could explore this for a better understanding of the interaction. Similarly, the current research did not explore sociodemographic and psychological determinants which also influence vaccine decisiveness. A study with a large sample of participants, which was conducted a few months after our data collection (November 2021 and January 2022), showed the interplay of sociodemographic, behavioral, and psychological factors influencing vaccine hesitancy in the general population and healthcare professionals in Cyprus [88] (see also [89]). The determinants found to influence higher vaccination rates in the general population included age (with older individuals being more likely to be vaccinated), higher trust in national healthcare authorities’ guidelines, and increased vaccination knowledge scores. Future research should consider the current findings in light of these variables for a more thorough understanding of the interplay of reasons that influence vaccine decisiveness. Other limitations to our study include the collection of data at a single point in time while the pandemic continues to evolve, not tracking the response rate to determine the number of participants clicking the link to take part in the study, and the use of self-report measures, which should be considered with discretion.

## 5. Conclusions

The current study contributes to a better understanding of the factors that influence COVID-19 vaccination decisiveness. Our results suggest that conspiracy theory belief is the critical factor that predicts vaccination decisiveness. Individuals who have high believability in conspiracy theories are more likely to be non-vaccinated. In addition, vaccinated individuals are more likely to believe that COVID-19 is a dangerous disease, indicating that the high perceived danger of COVID-19 positively affects vaccination uptake. Trust in science might increase the uptake of COVID-19 vaccines; the participants who trusted science were more likely to be vaccinated. Lastly, social media’s role was illustrated, i.e., individuals who use social media are more likely to believe in conspiracy theories. The findings from this study can be used by public health officials in their efforts to tackle vaccination hesitancy. Campaigns aiming to educate people about vaccine misinformation/conspiracy theories, improve health literacy, promote the public’s trust in science, and raise awareness about the danger of COVID-19 are highly recommended.

## Figures and Tables

**Table 1:** Participants’ demographics.

	Overall *n* (%)	Vaccinated *n* (%)	Non-Vaccinated *n* (%)
Vaccination Status		243 (66.90)	120 (33.10)
Age			
Young adults (18–30)	80 (22.04)	35 (43.75)	45 (56.25)
Early–middle-aged adults (31–50)	240 (66.12)	174 (72.50)	66 (27.50)
Late–middle-aged/older adults (51+)	43 (11.84)	34 (79.07)	9 (20.93)
Gender			
Females	267 (73.60)	180 (67.42)	87 (32.58)
Males	96 (26.40)	63 (65.63)	33 (34.37)
Place of Residency			
Urban	293 (80.70)	209 (71.33)	84 (28.67)
Rural	70 (19.30)	34 (48.57)	36 (51.43)
Education			
High-school diploma	39 (10.74)	24 (61.54)	15 (38.46)
Bachelor’s degree	137 (37.74)	85 (62.04)	52 (37.96)
Master’s and/or doctorate	187 (51.52)	134 (71.66)	53 (28.34)

**Table 2:** Mean response and percentage of no-to-weak, neutral, and moderate-to-strong belief for each conspiracy theory statement based on vaccination status.

How Strongly Do You Believe the Following Statements:	MEAN (SD)	SEM	Vaccination Status	% No-to-Weak Belief	% Neutral Belief	% Moderate-to-Strong Belief
1. COVID-19 doesn’t actually exist but is a plot to take away our personal freedom.	1.80 (1.039)	0.055	No	60.8	26.7	12.5
			Yes	84	11.9	4.1
2. Conventional drugs to fight the COVID-19 virus do not cure it but are a plot by big pharmaceutical companies. There is alternative medicine available (such as vitamins and herbs) that can cure or even prevent the COVID-19 virus.	2.40 (1.197)	0.063	No	29.2	39.2	31.7
			Yes	63.4	25.5	11.1
3. COVID-19 is no more dangerous than the flu, but the risks have been exaggerated as a way to restrict personal freedom.	2.48 (1.233)	0.065	No	30.8	30.8	38.3
			Yes	64.6	21	14.4
4. The mRNA technology used by some of the vaccines alters (can alter) our DNA.	2.41 (1.144)	0.060	No	29.2	34.2	36.7
			Yes	64.6	28.8	6.6
5. The COVID-19 vaccine can affect women’s fertility.	2.85 (1.075)	0.056	No	18.3	35.8	45.8
			Yes	42.8	42.8	14.4
6. You can get COVID-19 from the vaccine.	2.09 (1.151)	0.06	No	41.7	28.3	30
			Yes	77.4	16.5	6.2
7. Once I receive the COVID-19 vaccine I no longer need to wear a mask or take any precautions against the virus	2.05 (1.084)	0.057	No	64.2	24.2	11.7
			Yes	72.4	16.5	11.1
8. Researchers rushed the development of the COVID-19 vaccine so its effectiveness and safety cannot be trusted.	2.94 (1.135)	0.060	No	18.3	30	51.7
			Yes	44.0	34.6	21.4
9. The COVID-19 vaccine includes a tracking device or microchips.	1.53 (0.887)	0.047	No	68.3	21.7	10
			Yes	95.1	3.3	1.6
10. I have already been diagnosed with COVID-19 so I don’t need to receive the vaccine	1.95 (1.186)	0.062	No	53.3	24.2	22.5
			Yes	80.2	12.8	7.0

Note: mean on Likert scale 1–5 where 1 = “extremely unbelievable” to 5 = “extremely believable”; SEM = standard error of the mean; vaccination status, No = non-vaccinated, Yes = vaccinated; % no-to-weak belief = percentage of sample in 1st to 25th percentile; neutral belief = 26th to 75th percentile; and moderate-to-strong belief = 76th to 100th percentile.

**Table 3:** Logistic regression predicting the likelihood of vaccination decisiveness.

	B	S.E	Wald	df	*p*	Odds Ratio
Social media	−0.513	0.314	2.670	1	0.102	0.599
Conspiracy theory statements *	−0.131	0.027	23.945	1	<0.01	0.877
Trust in Science	0.006	0.016	0.164	1	0.685	1.006
Risk perception			5.602	2	0.061	
Risk perception (1)	0.132	0.605	0.047	1	0.828	1.141
Risk perception (2)	0.767	0.628	1.493	1	0.222	2.154
Constant	3.34	1.842	3.287	1	0.070	28.225

* *p* < 0.01.

## Data Availability

Data will be available upon reasonable request from the corresponding author.
